# Revisiting History Effects in Adverse-Pressure-Gradient Turbulent Boundary Layers

**DOI:** 10.1007/s10494-017-9845-7

**Published:** 2017-08-25

**Authors:** Ricardo Vinuesa, Ramis Örlü, Carlos Sanmiguel Vila, Andrea Ianiro, Stefano Discetti, Philipp Schlatter

**Affiliations:** 1 Linné FLOW Centre, KTH Mechanics, SE-100 44 Stockholm, Sweden; 2 Aerospace Engineering Group, Universidad Carlos III de Madrid, Leganés, Spain

**Keywords:** Turbulent boundary layer, Pressure gradient, Flow history, Numerical simulation

## Abstract

The goal of this study is to present a first step towards establishing criteria aimed at assessing whether a particular adverse-pressure-gradient (APG) turbulent boundary layer (TBL) can be considered *well-behaved*, i.e., whether it is independent of the inflow conditions and is exempt of numerical or experimental artifacts. To this end, we analyzed several high-quality datasets, including in-house numerical databases of APG TBLs developing over flat-plates and the suction side of a wing section, and five studies available in the literature. Due to the impact of the flow history on the particular state of the boundary layer, we developed three criteria of convergence to well-behaved conditions, to be used depending on the particular case under study. (i) In the first criterion, we develop empirical correlations defining the *R* *e*_*𝜃*_-evolution of the skin-friction coefficient and the shape factor in APG TBLs with constant values of the Clauser pressure-gradient parameter *β* = 1 and 2 (note that *β* = *δ* ^∗^/*τ*_*w*_d*P*_*e*_/d*x*, where *δ* ^∗^ is the displacement thickness, *τ*_*w*_ the wall-shear stress and d*P*_*e*_/d*x* the streamwise pressure gradient). (ii) In the second one, we propose a predictive method to obtain the skin-friction curve corresponding to an APG TBL subjected to any streamwise evolution of *β*, based only on data from zero-pressure-gradient TBLs. (iii) The third method relies on the diagnostic-plot concept modified with the shape factor, which scales APG TBLs subjected to a wide range of pressure-gradient conditions. These three criteria allow to ensure the correct flow development of a particular TBL, and thus to separate history and pressure-gradient effects in the analysis.

## Introduction

Fundamental studies of wall-bounded turbulence require accurate representations of the flow case under consideration. The main three *canonical* flow cases of wall-bounded turbulence, namely the zero-pressure-gradient (ZPG) turbulent boundary layer (TBL), the channel and the pipe, have the advantage of exhibiting simple, well-defined geometries and operating conditions. This allows to isolate the physics of wall-bounded turbulence from other effects present in more complicated configurations, such as for instance the secondary flows in ducted geometries [1]. It is essential to obtain reliable databases, where the flow case under study is independent of the inflow conditions and is exempt of numerical or experimental artifacts. In the context of the present work, we will denote such a flow case as *well-behaved*. Thus, a well-behaved ZPG experiment or simulation would be representative of a canonical ZPG TBL. Possible effects leading to deviations from the well-behaved state have received some attention in the recent years [2]. For instance, Marusic et al. [3] have assessed the impact of tripping devices on the development of TBLs in wind-tunnel experiments and Schlatter and Örlü [4] have investigated the effect of tripping in numerical simulations of wall-bounded turbulence. As stated by Chauhan et al. [5], among the three canonical cases stated above the ZPG TBL is the most challenging case to establish experimentally, due to its requirements in terms of tripping, a carefully-controlled pressure-gradient distribution, as well as the impact of local non-equilibrium conditions on the history of the flow. The fact that channels and pipes are fully-developed flows reduces slightly the complexity of the experimental setups, albeit a considerable development length is required (as well as a large aspect ratio in the channel case).

Motivated by the need to characterize well-behaved TBLs, Chauhan et al. [5] developed criteria based on the wake parameter π [6] and on the shape factor *H* = *δ* ^∗^/*𝜃* (where *δ* ^∗^ and *𝜃* denote the displacement and momentum thicknesses, respectively). They provided empirical relations describing the canonical ZPG TBL evolution of these parameters with Reynolds number, and established that the boundary layers satisfying such criteria exhibit inner-scaled mean velocity and defect profiles exempt of experimental artifacts. Note that the work by Chauhan et al. [5] expanded the previous studies by Coles [7] and Fernholz and Finley [8]. Schlatter and Örlü [9] showed that numerical ZPG TBLs can also be affected by the inflow conditions and the tripping method, and documented differences up to 5*%* in *H* among direct numerical simulation (DNS) databases, and interestingly up to 20*%* in the skin-friction coefficient *C*_*f*_ = 2(*u*_*τ*_/*U*_*e*_)^2^ (note that *U*_*e*_ is the velocity at the boundary-layer edge, and $u_{\tau }=\sqrt {\tau _{w}/ \rho }$ is the friction velocity; *τ*_*w*_ is the mean wall-shear stress and *ρ* is the fluid density). These differences, attributed to inflow and tripping conditions, were supported by a follow-up study [4], where it is shown that various ZPG TBLs would converge onto the same state in terms of integral quantities and turbulence statistics for Reynolds numbers based on momentum thickness *R* *e*_*𝜃*_ > 2000 if the tripping is performed at *R* *e*_*𝜃*_ < 300. Under these conditions, a particular boundary layer can be considered to be well-behaved, and therefore representative of a canonical ZPG TBL.

Marusic et al. [3] later considered three different tripping configurations in their wind-tunnel experiments. In addition to the standard sand-paper trip, which yields canonical ZPG TBL conditions, they also tested two threaded rods as tripping devices, which produce different levels of over-stimulation in the developing boundary layer. Based on a simplified approach of the work by Perry et al. [10], applied to the particular case of ZPG TBLs, they derived evolution equations to define the well-behaved development of the boundary layer, leading to curves relating *C*_*f*_, *H* and π, among others, with Reynolds number. They also documented the effect of the tripping, in particular the over-stimulation of the boundary layer, on the large-scale motions of the flow. Another relevant work where the effect of various tripping configurations, including cylindrical pins, distributed grit and wires, was considered is the study by Erm and Joubert [11].

It is also relevant to mention the recent work by Rodríguez-López et al. [12], who also analyzed various tripping conditions, in their case with the aim of achieving well-behaved high- *R* *e* TBLs. They used a sawtooth serrated fence, together with a number of arrays of cylinders in the spanwise direction, leading to various configurations with uniform and non-uniform blockage in the wall-normal direction. Their results show that when using tripping devices with uniform wall-normal blockage, it is possible to increase the momentum thickness of the TBL by 150% in comparison with standard tripping conditions, which effectively leads to the same increase in Reynolds number. Moreover, using tripping devices with non-uniform blockage in the wall-normal direction and 100% blockage at the wall leads to TBLs that are not well-behaved, even after very long distances downstream of the trip. They also identified the impact of the different trippings on the characteristics of the near-wall structures and their formation mechanisms, which explains the observed differences in the boundary layers.

As documented by Schlatter and Örlü [4], numerical databases are also subjected to effects related to the inflow conditions and the tripping mechanism, and it is therefore essential to ensure that the simulated flow is well-behaved. Regarding the impact of the mechanism used to generate the inflow conditions, it is interesting to highlight the difference between the approach by Schlatter and Örlü [4, 9], who considered a laminar boundary layer as inflow, and the method adopted by Sillero et al. [13], who generated synthetic inflow conditions based on the recycling method by Lund et al. [14]. One of the observations by Sillero et al. [13] was the fact that the turnover length might be the appropriate way of assessing whether the TBL was independent of the inflow or not when such synthetic conditions were used. Note that the turnover length is defined as the streamwise distance traveled by the eddies during a turnover time *δ*/*u*_*τ*_, where *δ* is the boundary-layer thickness. They also reported that quantities related to the large-scale motions of the flow required much longer distances to become independent of the inflow conditions, a conclusion that is in agreement with other studies such as the one by Schlatter and Örlü [4]. Another interesting numerical study is the work by Kozul et al. [15], who simulated a temporal boundary layer, and drew interesting connections between spatially- and temporally-developing boundary layers, as well as with experimental trip devices.

The aforementioned studies have highlighted the recent efforts with respect to the required development length in ZPG TBLs in order to establish well-behaved conditions. The situation for PG TBLs is even more complicated due to the additional effects of the local pressure gradient and of the pressure-gradient history. The practical relevance of TBLs under PG conditions explains the large interest, in particular when it comes to APG TBLs. Numerical simulations have been used to study the characteristics of PG TBLs, starting from the early work by Spalart and Watmuff [16], followed by the simulations by Skote et al. [17], and more recently by Lee and Sung [18] and Gungor et al. [19]. The effect of pressure gradient on the coherent structures in TBLs has been studied, among others, by Marquillie et al. [20] (who focused on near-wall streaks) and by Maciel et al. [21] (who analyzed two-point correlations of the streamwise velocity).

Based on wind-tunnel experiments, Sanmiguel Vila et al. [22] pointed out the fact that widely used methods to assess convergence towards well-behaved conditions rely on either accurate wall-shear stress measurements or full velocity profiles with accurate wall-position determination; or even on both. Since obtaining these quantities at a number of streamwise positions to assess such convergence in an experiment may be problematic, they introduced an alternative method based on the diagnostic-plot scaling [23]. The method relies on measurements of the streamwise mean velocity and local turbulence intensity within the outer region of the boundary layer, and does not require an accurate wall-position determination or knowledge of the friction velocity, therefore allowing to perform scans in the streamwise direction in order to assess with more robustness and less tedious procedures the region of convergence. Sanmiguel Vila et al. [22] evaluated their method with various tripping configurations, and also characterized the development length under such different inflow conditions in wind-tunnel experiments of ZPG TBLs. In the present article, we extend their work to adverse-pressure-gradient (APG) TBLs where, as discussed by Bobke et al. [24], the effect of the flow history is crucial, and develop the corresponding criteria to assess whether a particular TBL can be considered well-behaved or not. These criteria are, in particular, important in the case of wind-tunnel experiments, where the lack of accurate measurements everywhere in the domain of interest requires the use of such methods in order to ensure an adequate boundary-layer development.

As discussed below, in the present study we develop a number of criteria based on several in-house numerical databases, together with five high-quality sets of data available in the literature. In particular, we will consider the numerical work by Kitsios et al. [25], Lee [26] and Spalart and Watmuff [16], together with the experimental databases by Monty et al. [27] and Nagib et al. [28]. The various conditions present in the different databases lead to a variety of pressure-gradient evolutions, ranging from moderate-pressure-gradient cases to the strongly decelerated TBL on the suction side of a wing. The reason to limit the present work to these datasets is that not only the statistics at various streamwise positions (and hence Reynolds numbers) are required, but also their particular pressure-gradient history needs to be at hand. The latter was at our disposal only for some of the selected datasets. These data have allowed us to analyze the convergence to well-behaved conditions in scenarios with moderately complicated flow histories, and although some criteria are based on relatively low Reynolds-number ranges, the experimental measurements by Monty et al. [27] up to *R* *e*_*𝜃*_ = 18,700 and by Nagib et al. [28] up to *R* *e*_*𝜃*_ = 56,100 were used to establish the higher- *R* *e* behavior in one of the criteria discussed below.

It is important to stress that, unlike the ZPG TBL case, which is uniquely defined by the Reynolds number with a constant value of *β* = 0 (defined in Section 2), there are infinitely many possible realizations of well-behaved PG TBLs defined by their particular *β*(*x*) history and Reynolds number. In the present study we use a total of 11 databases of PG TBLs, which include cases with constant *β*, and relatively simple *β*(*x*) evolutions, including both increasing and decreasing *β* trends. Therefore, the criteria proposed in the present work are aimed at assessing whether a particular PG TBL is well-behaved or not, although their applicability is limited to the relatively simple *β*(*x*) configurations under study.

The article is structured as follows: in Section 2 we introduce the various in-house numerical databases under study in the present work and discuss their flow histories; we also introduce the five datasets from the literature analyzed in the following. In Section 3 we present convergence criteria based on empirical correlations for cases of constant and non-constant pressure-gradient magnitudes. In Section 4 we also present criteria for constant and non-constant pressure-gradient magnitudes, but in this case based on the diagnostic-plot scaling. Finally, in Section 5, we summarize the conclusions of the present work.

## Description of the Databases Analyzed in the Present Study

As discussed in the introduction, there are a number of criteria in the literature to assess the convergence of ZPG TBLs towards well-behaved conditions. Most of these criteria rely on empirical relations defining the evolution of the skin-friction coefficient, the shape factor or the wake parameter. However, it is more problematic to define criteria based on empirical correlations for general PG TBLs due to the effect of flow history, i.e., besides inflow and tripping as in the case of ZPG TBLs, also the additional dependence on the streamwise pressure-gradient history has to be taken into consideration [24]. The goal of the present study is to develop several convergence criteria for APG TBLs, extending some of the ideas and techniques previously developed for ZPG TBLs [22]. To this end, we will analyze several numerical and experimental databases of PG TBLs with very different flow histories. In Table 1 we summarize various in-house numerical databases analyzed in the present study. These include five flat-plate APG TBLs [24], all of them in what was denoted by Marusic et al. [29] as near-equilibrium conditions. These TBLs would only exhibit self-similarity at very high Reynolds numbers, and only in the outer region. As reported by Townsend [30] or Mellor and Gibson [31], this can be obtained when the freestream velocity *U*_*∞*_ is described by a power-law relation as *U*_*∞*_(*x*) = *C*(*x* − *x*_0_)^*m*^, where *x* is the streamwise coordinate, *x*_0_ is the power-law virtual origin, and *m* has to be larger than − 1/3 in order to obtain near-equilibrium conditions. All the flat-plate APG cases in Table 1 were defined with power-law freestream velocity distributions, and are therefore in near-equilibrium conditions. Note that additional discussions and interpretations regarding equilibrium in APG TBLs can be found in the studies reported in Refs. [32–34], in particular when it comes to scaling laws and their Reynolds-number evolution. Regarding the cases shown in Table 1, two of them exhibit long regions of constant values of the Clauser pressure-gradient parameter *β* = *δ* ^∗^/*τ*_*w*_d*P*_*e*_/d*x*, where d*P*_*e*_/d*x* is the streamwise pressure gradient. The constant- *β* cases are of great importance, since as discussed by Bobke et al. [24] they allow to study the effect of the pressure gradient on TBLs, isolating it from the effect of the flow history. In fact, the widely studied ZPG TBL is a particular case of near-equilibrium TBL with a constant value of *β* = 0. It could therefore be stated that an APG TBL is *canonical* when it is subjected to a constant value of *β*. Another in-house numerical database analyzed in the present work is the TBL developing on the suction side of a NACA4412 wing section reported by Hosseini et al. [35]. The interest of this latter flow case lies in the fact that the APG increases exponentially in the streamwise direction, and therefore the TBL is subjected to extreme pressure-gradient conditions, although it remains attached in the mean up to the trailing edge. Additional details regarding the numerical aspects are given in the respective references, but note that the flat-plate APG cases are obtained through well-resolved large-eddy simulations (LESs) using the Fourier–Chebyshev code SIMSON [36], and DNS was used for the wing, with the spectral-element code Nek5000 [37]. The DNS of ZPG TBL simulated by Schlatter and Örlü [9] with SIMSON is also included in Table 1, since it will be used in a number of comparisons in the present work.

**Table 1:** Summary of numerical databases analyzed in the present study, including the color that will be used to identify each cases throughout the article

Case	Range of *R* *e*_*𝜃*_ under study	Range of *β*	Color
*m*13	1610 < *R* *e*_*𝜃*_ < 3100	0.96 < *β* < 1.51	
*m*16	1740 < *R* *e*_*𝜃*_ < 3620	1.95 < *β* < 2.78	
*m*18	1750 < *R* *e*_*𝜃*_ < 4010	3.15 < *β* < 4.47	
*b*1	1470 < *R* *e*_*𝜃*_ < 2980	≃ 1	
*b*2	1760 < *R* *e*_*𝜃*_ < 3200	≃ 2	
Wing	750 < *R* *e*_*𝜃*_ < 2800	0.60 < *β* < 85	
ZPG	1000 < *R* *e*_*𝜃*_ < 4060	≃ 0	

Note that the setup corresponding to the *m*13, *m*16, *m*18 and the constant- *β* cases *b*1 and *b*2 is given by Bobke et al. [24]; the setup corresponding to the wing configuration can be found in Hosseini et al. [35]. The reference ZPG TBL data is reported by Schlatter and Örlü [9]

In order to develop robust criteria of convergence to well-behaved conditions, we also analyzed the five additional databases from the literature summarized in Table 2. In the DNS by Kitsios et al. [25] a region of constant *β* = 1 is established, over a higher Reynolds-number range than that of our *b*1 case shown in Table 1. Since both simulations are performed using high-order codes, their accuracy should be comparable, and therefore we will be able to define a more robust criterion for APG TBLs subjected to a constant value of *β* = 1, as discussed below. The DNS dataset by Lee [26] also includes APG TBLs with constant values of *β*, in their case equal to 0.73, 2.2 and 9. Note that the boundary-layer profiles for *β* ≃ 2.2 and 9 are located at the beginning of the constant- *β* region, and are therefore not considered in the following to establish constant- *β* criteria. However, since the *β*(*x*) evolution is reported, the results could be used to develop more robust criteria for flows with varying pressure-gradient magnitudes. With respect to the DNS TBL reported by Spalart and Watmuff [16], their flow is subjected to a pressure-gradient distribution ranging from a mild favorable pressure gradient (FPG) of *β* = −0.3 to a strong APG of *β* = 2. This database will be used in the determination of skin-friction criteria. Finally, the experimental profiles by Monty et al. [27] and Nagib et al. [28] span a higher Reynolds-number range than that of the other datasets, and therefore will be used to assess the high- *R* *e* trends in one of our criteria. However, due to the fact that the *β*(*x*) evolutions were not available in these cases, these databases can only be used in one of the criteria discussed below.

**Table 2:** Summary of additional databases available in the literature analyzed in the present study, including the symbol that will be used to identify them throughout the article

Reference	Type of data	Range of *R* *e*_*𝜃*_ under study	Range of *β*	Symbol
Kitsios et al. [25]	DNS	3500 < *R* *e*_*𝜃*_ < 4800	≃ 1	
Lee [26]	DNS	1605 < *R* *e*_*𝜃*_ < 2840	≃ 0.73, ≃ 2.2 and ≃ 9	
Spalart and Watmuff [16]	DNS	640 < *R* *e*_*𝜃*_ < 1600	− 0.3 < *β* < 2	
Monty et al. [27]	Experiment	6100 < *R* *e*_*𝜃*_ < 18700	0.91 < *β* < 4.73	
Nagib et al. [28]	Experiment	11600 < *R* *e*_*𝜃*_ < 56100	− 0.2 < *β* < 0.3	

### Characterization of the in-house APG TBLs

**Fig. 1:**
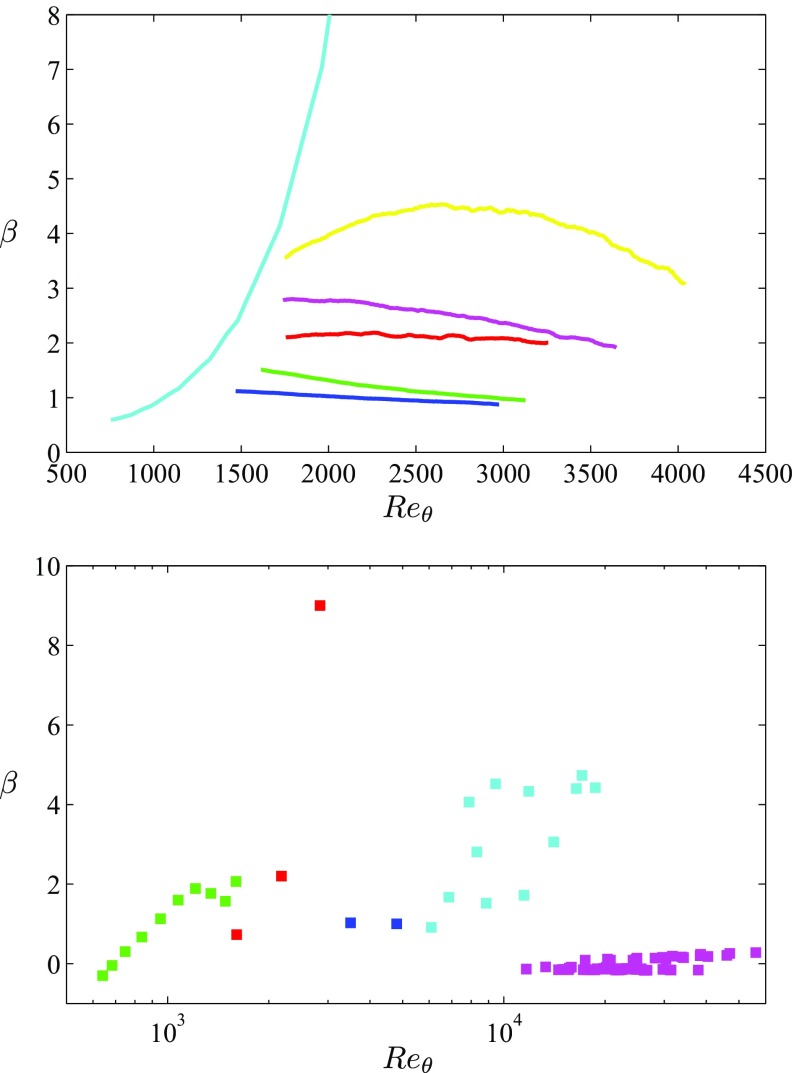
Evolution of the Clauser pressure-gradient parameter with *R* *e*_*𝜃*_ for (top) the various in-house APG cases, and (bottom) five additional databases available in the literature. Colors and symbols given in Tables 1 and 2 (color online)

The in-house numerical databases introduced in Section 2 and summarized in Table 1 are briefly characterized here. In Fig. 1 (top) we show the evolution of the Clauser pressure-gradient parameter *β* with *R* *e*_*𝜃*_ for the various APG cases under consideration. The first observation is the fact that the *b*1 and *b*2 cases exhibit long regions of constant values of *β*, with average values of *β* = 1.0 and *β* = 2.1, respectively. Note that positive values of *β* are associated with APGs, i.e., with decelerated boundary layers. This deceleration produces an increase in the wall-normal velocity, which in turn leads to larger boundary-layer thicknesses in APG TBLs. As argued, among others, by Bobke et al. [24] and discussed in Section 3.2, the state of a particular APG TBL is not uniquely determined by the Reynolds number and the local value of *β*, but also by its pressure-gradient history *β*(*x*). The *m*13 and *m*16 cases, which constitute also near-equilibrium APG TBLs, exhibit a slightly decreasing trend in *β*. They start from stronger APG conditions than the *b*1 and *b*2 configurations, and progressively reach very similar pressure-gradient magnitudes. On the other hand, the stronger flat-plate APG case *m*18, starts with an increasing *β* trend from around 3.5 up to approximately 4.5 (a value of *β* achieved at *R* *e*_*𝜃*_ ≃ 2700). Beyond this point, it also exhibits a decreasing trend in *β*, with a final value of around 3.2 (corresponding to strong APG conditions), at *R* *e*_*𝜃*_ ≃ 4010. The *β* in the wing shows an exponential increase as *R* *e*_*𝜃*_ increases, a trend significantly different from that exhibited by the near-equilibrium boundary layers. Note that although a very large value of *β* ≃ 85 is observed close to the trailing edge of the wing, the boundary layer on the suction side only exhibits up to around 30*%* instantaneous reversed flow [38], and the boundary layer remains attached in the mean. Figure 1 (bottom) includes, for reference, the Reynolds-number evolution of the Clauser pressure-gradient parameter of the five databases from the literature listed in Table 2. Note that in the two databases containing regions of constant *β*, namely the ones by Kitsios et al. [25] and Lee [26], only the *β* values from the profiles analyzed here are shown.

**Fig. 2:**
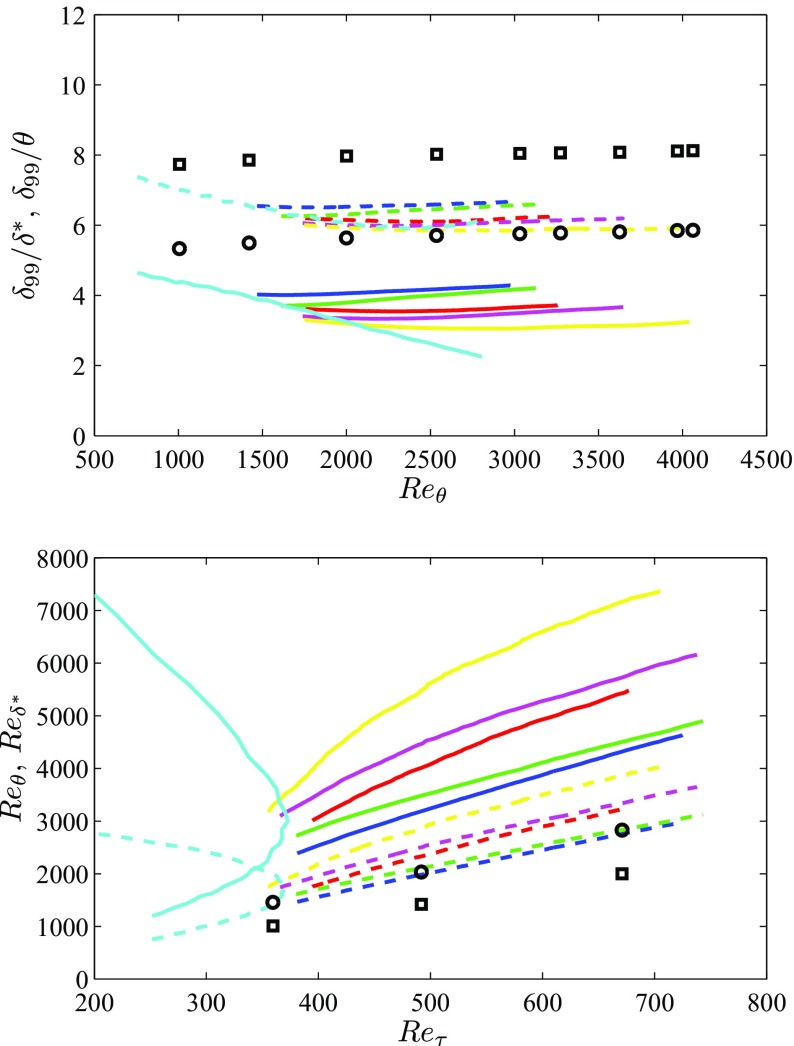
(Top) Evolution of the ratio between boundary-layer thickness and  displacement and  momentum thickness with *R* *e*_*𝜃*_ for the various APG cases. (Bottom) Reynolds number based on  displacement and  momentum thickness for the various APG cases, as a function of friction Reynolds number. The colors are given in Table 1, and the ZPG results are represented by (∘) and ($\Box $) for displacement and momentum thickness, respectively (color online)

The different development of the boundary layers are illustrated in Fig. 2, where the various boundary-layer thicknesses are documented. As discussed by Vinuesa et al. [39], the boundary-layer thickness is a rather ambiguous quantity in APG TBLs compared to the ZPG case. This is due to the fact that, in the former, the streamwise velocity is not necessarily constant beyond the boundary-layer edge, a fact that explains some of the irregularities observed in the boundary-layer parameters shown in Figs. 1 and 2. In the present work, the 99*%* boundary-layer thickness *δ*_99_ is calculated following the procedure by Vinuesa et al. [39], which is based on the diagnostic-plot concept by Alfredsson et al. [23]. In Fig. 2 (top) we show the evolution of the ratios *δ*_99_/*δ* ^∗^ and *δ*_99_/*𝜃*, which are sensitive indicators of the boundary-layer growth [4]. Lower values of these ratios are associated with velocity profiles exhibiting lower velocities in the near-wall region, as it is the case in APGs due to the increased wall-normal momentum transfer. As expected, the values of *δ*_99_/*δ* ^∗^ from the APG cases are below the ones of the ZPG, with the ratio decreasing for increasing values of *β*. Interestingly, cases *b*1 and *b*2 show a slightly increasing behavior, approximately parallel to the trend from the ZPG boundary layer. The rest of the flat-plate cases also show a slightly increasing trend, although not parallel to the one described by the ZPG TBL case. Moreover, the *m*18 boundary layer, with the strongest APG magnitude, shows almost no growth for *R* *e*_*𝜃*_ > 3500. On the other hand, the APG on the suction side of the wing shows a decreasing trend throughout the whole domain of interest, a manifestation of the progressively stronger APG conditions it is subjected to, and connected to the fact that this boundary layer is clearly not in near-equilibrium conditions. Also note that the APG increases the thickness of the boundary layer, which explains the larger values of *δ* ^∗^ at higher *β*. Similar conclusions can be drawn from the *δ*_99_/*𝜃* ratios, also presented in the same figure.

The Reynolds number based on momentum and displacement thickness is shown, as a function of the friction Reynolds number *R* *e*_*τ*_ = *δ*_99_ *u*_*τ*_/*ν* (where *ν* is the fluid kinematic viscosity), in Fig. 2 (bottom) for all the cases. In this figure it can be observed that stronger APGs lead to higher values of $Re_{\delta ^{*}}$ and *R* *e*_*𝜃*_. Note that these Reynolds numbers are formed with the local values of the the edge velocity *U*_*e*_ and either *δ* ^∗^ or *𝜃*. This implies that although *U*_*e*_ decreases in APG TBLs due to the streamwise deceleration, the increase in *δ* ^∗^ and *𝜃* is larger than the velocity decrease, thus yielding a significant increase in both Reynolds numbers for progressively stronger APG conditions. The *b*1 and *b*2 cases also show trends approximately parallel to the ZPG ones (except the lower- *R* *e* range in the *β* = 2 case, which exhibits smaller values of $Re_{\delta ^{*}}$), and as expected the *m*18 configuration shows the largest values among the flat-plate cases. The APG boundary layer on the suction side of the wing section exhibits an interesting trend, with progressively increasing values of $Re_{\delta ^{*}}$ and *R* *e*_*𝜃*_ in the streamwise direction, but with a maximum value of *R* *e*_*τ*_ ≃ 373. This value is reached at a streamwise distance of 80*%* of the chord length on the suction side of the wing, and corresponds to $Re_{\delta ^{*}}=2990$ and *R* *e*_*𝜃*_ = 1720. This trend in *R* *e*_*τ*_ is produced by the progressively increasing APG magnitude, which leads to an increase in *δ*_99_, but also to a decrease in the friction velocity *u*_*τ*_. At 80*%* of the chord length the decrease in *u*_*τ*_ overcomes the increase in *δ*_99_, a fact that leads to the decrease in *R* *e*_*τ*_. This also shows that *R* *e*_*τ*_ is not a good quantity to study the asymptotic behavior of strongly decelerated APG TBLs, and $Re_{\delta ^{*}}$ or *R* *e*_*𝜃*_ would be preferable instead as shown in Fig. 2 (bottom). On the other hand, the parallel evolution of *R* *e*_*𝜃*_ and $Re_{\delta ^{*}}$ with *R* *e*_*τ*_ for the flat-plate boundary layers studied here, indicates that for the mild APG conditions investigated here, any of the aforementioned Reynolds numbers is appropriate to study Reynolds-number effects. For the following discussion, which includes the APG TBL on the suction side of the wing, the momentum-loss Reynolds number *R* *e*_*𝜃*_ is preferred.

After discussing the development of the various APG TBLs under study, it is clear that the very different flow histories (defined by the various *β* distributions in Fig. 1) lead to significant differences in the evolution of the boundary layers. In Sections 3 and 4 we will discuss the possibilities to define criteria to discern whether an APG TBL can be considered as well-behaved or not, using both the mentioned in-house databases and the five additional datasets from the literature summarized in Table 2.

## Criteria to Identify “Well-behaved” APG TBLs

### Cases with constant *β*

In the introduction we discussed the importance of developing criteria to ensure that the flow cases under investigation can be considered to be well-behaved, i.e., independent of the inflow conditions and exempt of any numerical or experimental artifacts. Chauhan et al. [5] proposed empirical correlations describing the *R* *e*-evolution of the shape factor and the wake parameter, in the context of ZPG TBLs. The advantage of the ZPG configuration is the fact that the well-behaved cases are not affected by (streamwise pressure-gradient) history effects, since this boundary layer is defined by a constant value of *β* = 0. Therefore, any deviation from the proposed empirical correlations can be attributed to local non-equilibrium effects or problems with the development of the boundary layer (e.g. due to strong over- or under-tripping), which would basically imply that the particular flow case cannot be considered to be a canonical ZPG TBL. Nevertheless, general PG TBLs are greatly affected by history effects, as can be observed in Figs. 1 and 2. This adds some difficulty when establishing criteria of convergence towards well-behaved conditions. There would be as many well-behaved evolutions of *H*, π or *C*_*f*_ as possible pressure-gradient histories, and therefore it is not possible to use such criteria for general PG TBLs. It is also interesting to note that the *b*1 and *b*2 cases, with constant value of *β*, are subjected to the same well-defined pressure-gradient magnitude throughout a considerable streamwise extent. In the present section we will focus on the *b*1 and *b*2 cases, together with the APG TBL with constant value of *β* = 1 by Kitsios et al. [25], and report empirical curves that can be used to assess whether a particular APG TBL, with constant *β* = 1 or 2, can be considered to be well-behaved. Note that given the relatively low Reynolds number available for the present databases, we will focus on the skin-friction coefficient and shape factor, and therefore we will not provide an empirical curve for the wake parameter, which would require higher Reynolds numbers in order to not be affected by low- *R* *e* effects.

The skin-friction coefficient *C*_*f*_ can be written as follows, assuming that the overlap region follows the logarithmic law [40]:

1 $$ C_{f}=2 \left[ \frac{1}{\kappa} \ln \left( Re_{\theta} \right) +C \right]^{-2}, $$

where *κ* is the von Kármán coefficient, and *C* is a constant. As discussed by Chauhan et al. [5], this skin-friction relation is the leading-order term of a high- *R* *e* expansion of *C*_*f*_, and higher-order terms can be added to account for low- *R* *e* effects. Although Chauhan et al. [5] expressed *C*_*f*_ in terms of $Re_{\delta ^{*}}$, here we express it in terms of *R* *e*_*𝜃*_ and retain the same higher-order terms, leading to the following relation:

2 $$ C_{f}=2 \left[ \frac{1}{\kappa} \ln \left( Re_{\theta} \right) +C + \frac{ D_{0} \ln \left( Re_{\theta} \right)}{Re_{\theta}} + \frac{D_{1}}{Re_{\theta}} \right]^{-2}, $$

where *D*_0_ and *D*_1_ are also constants. Note that the logarithmic skin-friction relation and its low- *R* *e* correction have found widespread preference compared to other empirical relations since the early work by Rotta [41]. The values of the various coefficients in the correlations (1) and (2) used in the present work for the ZPG, and the APG cases with constant *β* = 1 (including Kitsios et al. [25]) and 2, are given in Table 3. In order to obtain the various coefficients, the data with *R* *e*_*𝜃*_ > 2000 was first fit to Eq. 1, which led to the values of *κ* and *C*. Then, the full *R* *e*-range was fit to Eq. 2, using the previously obtained values of *κ* and *C*, to determine the coefficients in the higher-order terms. The ZPG values *κ* = 0.384 and *C* = 4.127 were also reported by Nagib et al. [40], and the coefficients *D*_0_ and *D*_1_ are of the same order as the ones obtained by Chauhan et al. [5] in their $Re_{\delta ^{*}}$-based correlation. Regarding the APG cases, note that the values of *κ* decrease with increasing APG magnitude, as reported for instance by Nagib and Chauhan [42] or Nickels [43].

**Table 3:** Summary of coefficients used in the *C*_*f*_ and *H* correlations (1)–(4), for the various cases in the present study

Case	*κ*	*C*	*D*_0_	*D*_1_	*C* ^′^	*E*_1_
ZPG	0.384	4.127	220	− 1945	7.135	−19.12
APG with constant *β* = 1	0.361	5.300	250	− 2100	9.932	− 2.415
APG with constant *β* = 2	0.349	6.886	260	− 2500	12.53	−88.41

Using the definitions of *δ* ^∗^ and *𝜃* it is possible to derive the following equation for the shape factor *H* [44]:

3 $$ H=\frac{1}{1-\left( C^{\prime} / U^{+}_{e} \right)}, $$

where $U^{+}_{e}=U_{e}/u_{\tau }$ is the inner-scaled boundary-layer edge velocity, $C^{\prime }={\int }_{0}^{\infty } W^{+2} \ \mathrm {d}\left (y / {\Delta } \right )$, $W^{+}=U^{+}_{e}-U^{+}$ and ${\Delta }=U^{+}_{e} \delta ^{*}$ is the Rotta–Clauser length scale. As in the case of Eq. 1, it is possible to extend this relation to include low- *R* *e* effects by considering an additional term of *O*(1/*R* *e*_*𝜃*_), similarly to what was done by Chauhan et al. [5] with $Re_{\delta ^{*}}$, as follows:

4 $$ H=\frac{1}{1-\left( C^{\prime} / U^{+}_{e} \right)} + \frac{E_{1}}{Re_{\theta}}, $$

where *E*_1_ is a constant. The values used in the present work for the different cases in Eqs. 3 and 4 are also summarized in Table 3. Note that, as in the *C*_*f*_ correlations, the *C* ^′^ value was obtained by fitting the data with *R* *e*_*𝜃*_ > 2000 to Eq. 3, and then using that value to obtain the higher-order coefficient *E*_1_ from the complete dataset using (4). Moreover, the inner-scaled edge velocity was obtained from Eq. 1, through the relation $U^{+}_{e}=\sqrt {2/C_{f}}$. Interestingly, the *b*1 and Kitsios et al. [25] cases with *β* = 1 led to a very small value of *E*_1_ = −2.415, which means that the high- *R* *e* version of the *H* correlation is almost capable of representing the complete Reynolds-number range. Note that the value from the ZPG case *C* ^′^ = 7.135 was also reported by Nagib et al. [40], and that the values of *C* ^′^ from the cases with constant *β* = 1 and 2, are 9.932 and 12.53 respectively, are close to the values obtained by integrating the inner-scaled mean velocity defect profiles (with average values over the datasets of 9.87 and 12.09, respectively). The small deviations between the *C* ^′^ values obtained from the curve fit to *H* and from the individual velocity profiles can be associated to low- *R* *e* effects.

**Fig. 3:**
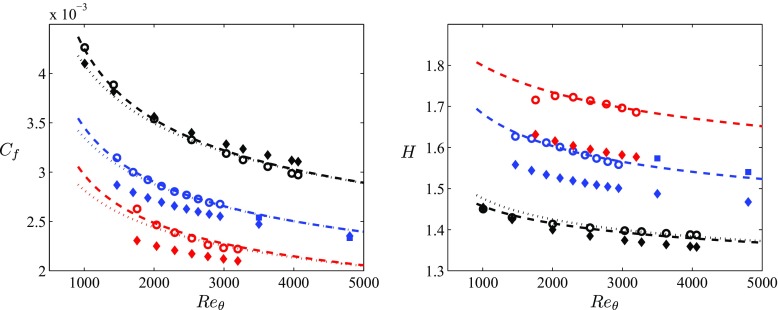
(Left) Evolution of the skin-friction coefficient with Reynolds number based on momentum thickness. (∘) represents values from the individual profiles corresponding to the various in-house simulations,  corresponds to the high- *R* *e* correlation (1) and  to the low- *R* *e* curve given by Eq. 2. (Right) Evolution with *R* *e*_*𝜃*_ of the shape factor, where again (∘) represent values from individual profiles,  corresponds to Eq. 3 and  to Eq. 4. Colors represent the constant- *β* cases defined in Table 1. As shown in Table 2,  corresponds to data from Kitsios et al. [25]. Predictions from the correlations by Mellor and Gibson [31], at the same Reynolds numbers as those of the cases presented here, are represented by  and the colors are according to the cases described in Table 1 (color online)

Figure 3 shows the boundary-layer parameters in terms of *R* *e*_*𝜃*_, compared with the empirical correlations (1)–(4). In Fig. 3 (left) we show the skin-friction coefficient *C*_*f*_, which as expected exhibits values progressively smaller as the APG magnitude increases. This figure shows that the evolution with *R* *e*_*𝜃*_ of the skin-friction coefficients from the constant *β* = 0, 1 and 2 cases are in very good agreement with Eq. 1 for *R* *e*_*𝜃*_ > 2000, thus providing a possible criterion to assess whether a particular boundary layer exhibits well-behaved conditions beyond this Reynolds number. It can also be observed that using the low- *R* *e* correction in Eq. 2, the complete dataset is well reproduced by the empirical relation, and therefore this constitutes a more complete criterion of convergence to well-behaved conditions. Regarding the evolution of the shape factor shown in Fig. 3 (right), it can be observed that stronger APG boundary layers exhibit larger values of *H*. This is due to the fact that the APG increases the thickness of the boundary layer, with the consequent reduction in wall-shear stress. The high- *R* *e* correlation (3) shown for the ZPG case again represents the data well for *R* *e*_*𝜃*_ > 2000, although the low- *R* *e* correction improves the agreement with the numerical data below this Reynolds number. Note that for the cases with constant *β* = 1 and 2 we only show the correlation with the low- *R* *e* correction (4), which represents very well the full range of data, with the exception of the lowest Reynolds number in the *b*2 case. The results presented here show that the empirical correlations (2) and Eq. 4, for *C*_*f*_ and *H* respectively, can be used as convergence criteria in APG TBLs with constant values of *β* = 1 and 2. This is an important conclusion, because it also highlights the fact that it is possible to define specific APG conditions in which the TBL is subjected to a constant magnitude of the pressure gradient, analogously to ZPG TBL cases.

The interesting theoretical work by Mellor and Gibson [31] also led to expressions for the skin-friction coefficient and the shape factor in PG TBLs subjected to constant values of *β*. In particular, they proposed a skin-friction relation of the same type as Eq. 1, defined in terms of $Re_{\delta ^{*}}$ instead of *R* *e*_*𝜃*_. However, the most relevant difference with respect to the present study is the fact that they proposed a constant value of *κ* = 0.41 regardless of the pressure-gradient magnitude, and a value of *C* related to the particular *β*. Thus, in their analysis they considered the slope of the logarithmic region to be independent of the pressure gradient, with different intercepts according to the PG magnitude. Predictions from the skin-friction relation proposed by Mellor and Gibson [31] at the same Reynolds numbers as the ones discussed for the *β* = 0, 1 and 2 cases are shown in Fig. 3 (left). Note that although the results are shown in terms of *R* *e*_*𝜃*_, the corresponding $Re_{\delta ^{*}}$ values were used to calculate *C*_*f*_. Due to the fact that it is necessary to have the relation between $Re_{\delta ^{*}}$ and *R* *e*_*𝜃*_ (i.e., the shape factor) to compare the skin-friction relation proposed in the present study and the one by Mellor and Gibson [31], we only show the predictions from the latter over the Reynolds-number range spanned by the available constant- *β* datasets. Firstly, note that the value of *κ* used by Mellor and Gibson [31], even for the ZPG case, is higher than the value of *κ* = 0.384 recently reported by Nagib et al. [40] among others. This could explain the small deviations between our ZPG data and the correlation by Mellor and Gibson [31]. At low Reynolds number the Mellor and Gibson [31] correlation is in good agreement with Eq. 1, i.e., our skin-friction correlation for ZPG TBLs without the low- *R* *e* correction. At higher Reynolds numbers the correlation from Mellor and Gibson [31] slightly overestimates the skin-friction trend. On the other hand, in the *β* = 1 and 2 cases the correlation from Mellor and Gibson [31] underestimates the skin-friction trends from the *b*1 and *b*2 cases, up to *R* *e*_*𝜃*_ values of around 3000. However, the *C*_*f*_ predictions from Mellor and Gibson [31] for *β* = 1 at higher Reynolds numbers are in very good agreement with the values reported by Kitsios et al. [25]. Due to the fact that we do not have *H* values beyond this Reynolds number, we cannot predict whether the curve from Mellor and Gibson [31] would be in good agreement with Eq. 2 at higher Reynolds numbers for *β* = 1. Nevertheless, it appears that the use of a constant value of *κ* for the whole pressure-gradient range, as it is the case of the correlation by Mellor and Gibson [31], could lead to inaccuracies in boundary-layer predictions as reported, for instance, by Nagib and Chauhan [42] or Vinuesa et al. [45]. In this context, Dixit and Ramesh [46] developed a modified Clauser-chart method to predict the wall-shear stress in PG TBLs, based on variable values of *κ* according to the pressure-gradient conditions. It is also important to mention that in the definition of the freestream boundary condition from the simulation by Kitsios et al. [25], the evolution of the displacement thickness in the streamwise direction is prescribed according to the work by Mellor and Gibson [31]. However, an assessment of the impact of this on the results is beyond the scope of the present work.

In addition to the skin-friction correlation, Mellor and Gibson [31] also provided the theoretical background to estimate the Reynolds-number evolution of the shape factor *H* in PG TBLs subjected to constant values of *β*. In particular, they provided the evolution of the defect shape factor *G* with *β*, a quantity that can then be used to calculate the shape factor through the relation: $G= \left (H -1 \right ) / \left (H \sqrt {C_{f}/2} \right )$. Since this quantity relies on *C*_*f*_, it can be expressed in terms of $Re_{\delta ^{*}}$ and then represented as a function of *R* *e*_*𝜃*_ as shown in Fig. 3 (right). Note that Mellor and Gibson [31] introduced an additional Reynolds-number correction for *G*, which was also considered in the present study. In this figure it can be observed that although the shape-factor equation by Mellor and Gibson [31] is in good agreement with the low- *R* *e* ZPG data, it underestimates the values of *H* at higher Reynolds numbers. Regarding the *β* = 1 and 2 cases, the trends predicted by Mellor and Gibson [31] are below the expected values, a deviation that appears to increase at higher values of *β*. This discrepancy could again be connected to the fact that a constant value of *κ* is considered in the log-law description over the whole pressure-gradient range.

### Cases with arbitrary *β*(*Re*_*𝜃*_) distributions

**Fig. 4:**
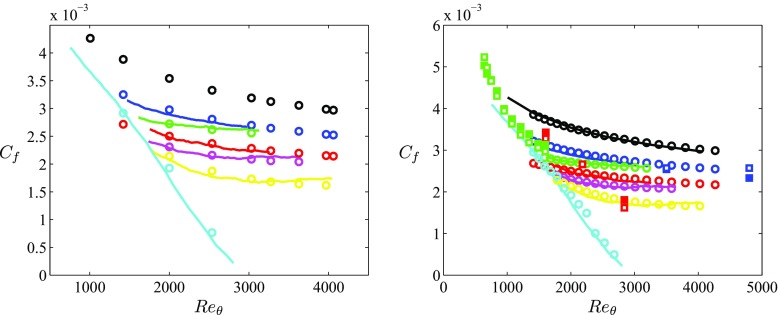
(Left) Evolution of the skin-friction coefficient with *R* *e*_*𝜃*_, where the colored  represent the various in-house APG cases, and the black (∘) the ZPG curve. Colored (∘) represent estimations of the *C*_*f*_ values from the various APG boundary layers using Eq. 8, and the ZPG DNS data. (Right) Evolution of the skin-friction coefficient with *R* *e*_*𝜃*_, where the colored  represent the various in-house boundary-layer cases, including the ZPG. Colored (∘) represent estimations of *C*_*f*_ using Eq. 8, evaluated based on the ZPG correlations (2) and (4). Note that the DNS data is not used in this panel, and that although the ZPG correlations are continuous, the values of $\overline {\beta }$ are evaluated at the discrete set of *R* *e*_*𝜃*_ values indicated by (∘). Colors given in Table 1. Filled  correspond to the cases summarized in Table 2, whereas empty $(\Box )$ denotes estimations of *C*_*f*_ using Eq. 8, together with the ZPG correlations (2) and (4) for the same cases (color online)

Although the convergence criteria given by Eqs. 2 and 4 are useful when used for APG TBLs with constant *β*, it is not practical to define a different criterion for each possible *β*(*R* *e*_*𝜃*_) history. On the other hand, it would be interesting to have a criterion to be used for general APG TBLs. In Fig. 4 (left) we show the skin-friction curves from all the in-house APG boundary layers in the present study, together with the ZPG case. As observed in Figs. 3 (left) and 4 (left), the *C*_*f*_ curves of the *b*1 and *b*2 cases are similar to the one of the ZPG TBL, although shifted towards lower wall-shear values for increasing *β*. In particular, Fig. 4 (left) shows that the *C*_*f*_ curves from the constant- *β* cases can be reproduced empirically from the ZPG DNS data as follows:

5 $$ C_{f,b1} \approx \frac{C_{f,\text{ZPG}}}{H_{\text{ZPG}}^{0.5}}, $$

6 $$ C_{f,b2} \approx \frac{C_{f,\text{ZPG}}}{H_{\text{ZPG}}}, $$

where *C*_*f*,*b*1_ and *C*_*f*,*b*2_ are the skin-friction coefficients from the *b*1 and *b*2 cases, and *C*_*f*,ZPG_ and *H*_ZPG_ are the skin-friction coefficient and shape factor from the ZPG DNS, all of them at the same value of *R* *e*_*𝜃*_. This result can be generalized to constant- *β* cases in the moderate range of pressure-gradient magnitudes investigated in the present study as follows:

7 $$ C_{f,\beta}=\frac{C_{f,\text{ZPG}}}{H_{\text{ZPG}}^{\beta / 2}}, $$

where *C*_*f*,*β*_ is the skin-friction coefficient of an APG TBL with a constant value of *β*. This is a very interesting result, because it implies that it appears possible to reproduce skin-friction curves from constant- *β* APG TBLs, based uniquely on ZPG TBL data. In fact, since the estimation of *C*_*f*,*β*_ is done at the same *R* *e*_*𝜃*_ as the ZPG TBL, Eq. 7 can be interpreted as a “correction” to the ZPG trend, through the shape factor (which increases with the APG) and the pressure-gradient magnitude. Moreover, in Fig. 4 (left) we also show that it is possible to reproduce the skin-friction curve of an APG TBL with a certain *β*(*R* *e*_*𝜃*_) distribution based only on ZPG DNS results as follows:

8 $$ C_{f,\text{APG}}=\frac{C_{f,\text{ZPG}}}{H_{\text{ZPG}}^{\overline{\beta}/2}}, $$

where *C*_*f*,APG_ is the skin-friction coefficient of an APG TBL subjected to a known *β*(*R* *e*_*𝜃*_) pressure-gradient distribution. In order to incorporate the effect of the flow history into this relation, we used the averaged $\overline {\beta }$, calculated as:

9 $$ \overline{\beta}(Re_{\theta})=\frac{1}{Re_{\theta}-Re_{\theta,0}} {\int}_{Re_{\theta,0}}^{Re_{\theta}} \beta(Re_{\theta}) \ \mathrm{d}Re_{\theta}, $$

where $\overline {\beta }(Re_{\theta })$ represents the average value of *β* up to a certain Reynolds number. Note that *R* *e*_*𝜃*,0_ is the point where the averaging is started, and in the case of the results in Fig. 4 it was set to the first available *R* *e*_*𝜃*_ value in the dataset above 1000. Figure 4 (left) reflects a quite remarkable agreement, i.e., it is possible to reproduce the *C*_*f*_ curves from all the APG TBLs under consideration in this work, using only the ZPG TBL DNS data and the *β* evolution. The flow histories from the various APG cases are encapsulated in the $\overline {\beta }$ parameter, calculated through relation (9). Note that not only the *C*_*f*_ curves from the moderately complicated flow histories in the three near-equilibrium flat-plate cases (*m*13, *m*16 and *m*18) are very well reproduced in Fig. 4 (left), but also the extreme pressure-gradient distribution exhibited by the wing profile (see Fig. 1). Ignoring the pressure-gradient history by utilizing the local value of *β* instead would result in curves that deviate up to 100*%* with respect to the reference data in the wing case. It is important to note that the results shown in Figure 4 (left) were obtained using the ZPG DNS data, at the particular *R* *e*_*𝜃*_ values where those profiles are available. Moreover, in Fig. 4 (right) we test the possibility of reproducing the *C*_*f*_ curve of any APG TBL using the ZPG correlations for *C*_*f*_ and *H*, i.e., relations (2) and (4), respectively. Using the correlations instead of the DNS data significantly extends the applicability of the method proposed here. Interestingly, the *C*_*f*_ curves estimated using the ZPG correlations of *C*_*f*_ and *H* also agree very well with the reference in-house cases, as observed in Fig. 4 (right). Note, however, the slight over-prediction of the *C*_*f*_ curve from the wing at around *R* *e*_*𝜃*_ ≃ 2000. In addition to the fact that the method proposed above is only based on empirical observations, other additional aspects may contribute to this. Firstly, small inaccuracies in the ZPG correlations around this Reynolds number, magnified by the larger pressure-gradient magnitude in comparison with the flat-plate APGs. A second factor that could explain this small over-prediction comes from the method used to calculate the average *β*, which essentially integrates in *R* *e*_*𝜃*_ to account for the history effects. Further work is required to develop a more sophisticated method to incorporate the history, possibly performing some windowing technique in order to attribute a larger weight to the local value of *β*.

This method is further tested in Fig. 4 (right) with the additional numerical databases by Spalart and Watmuff [16], Kitsios et al. [25] and Lee [26]. The APG TBL by Kitsios et al. [25] is subjected to a constant value of *β* = 1 and, as can be observed in Fig. 4 (right), the agreement with the predictions from Eq. 8 is very good. This equation is also able to capture the three points extracted from the three constant- *β* APG TBLs by Lee [26], which span a lower Reynolds-number range but a wider range of *β* values up to 9. Moreover, the skin-friction coefficient from the boundary layer by Spalart and Watmuff [16], which is in fact subjected to a more complex pressure-gradient history (starting with a mild FPG leading to a strong APG), is also well reproduced by Eq. 8. Note that in this case, the negative values of *β* would lead to a skin-friction coefficient larger than the one of a ZPG TBL (as expected for accelerated boundary layers), a fact that is also reproduced by Eq. 8. Therefore, the method proposed here to estimate the skin-friction coefficient provides very good agreement with the PG TBL results, over a wide range of pressure-gradient conditions. The only knowledge required to obtain the *C*_*f*_ curve is the *β*(*R* *e*_*𝜃*_) distribution of the APG boundary layer. Then, the ZPG correlations (2) and (4) can be used to construct a reference skin-friction curve for any APG TBL (as well as several mild FPG cases, which were well reproduced by this method).

## Convergence Criteria Based on the Diagnostic-Plot Scaling

### Cases with arbitrary *β*(*Re*_*𝜃*_) distributions

In Section 3.1 we discussed criteria to assess the convergence to well-behaved conditions of constant- *β* TBLs, based on empirical curves for *C*_*f*_ and *H*. Then, a criterion based on *C*_*f*_ for general APG TBLs with any *β* distribution was presented in Section 3.2. Although the criteria based on *C*_*f*_ gives a good indication of whether a particular boundary-layer profile can be considered well-behaved or not, if the goal is to assess the location after which the flow exhibits well-behaved conditions in a wind-tunnel experiment then these criteria are not practical. This is due to the fact that in order to accurately determine the wall-shear stress experimentally it is necessary to perform direct measurements, using for instance the oil-film interferometry (OFI) technique described, for example, by Vinuesa and Örlü [47]. It is usually not possible to perform OFI measurements at a large number of streamwise locations, and therefore the *C*_*f*_ criteria is not suitable to assess the location of convergence to well-behaved conditions.

**Fig. 5:**
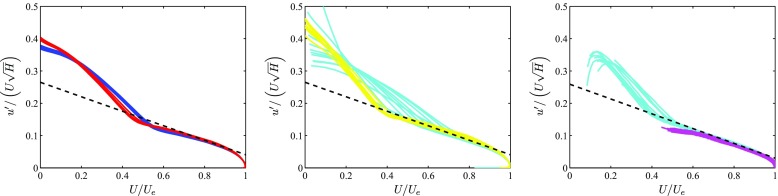
Diagnostic-plot scaling modified with the shape factor *H*, applied to several PG TBL cases. Colors are given in Table 1 for the in-house databases shown in (left) and (middle), whereas the colors from the high- *R* *e* cases shown in (right) are given in Table 2.  represents the linear relation (11). In (left) and (middle), the values *α*_*H*_ = 0.264 and *β*_*H*_ = 0.227 (obtained from Eqs. 12 and 13 at *R* *e*_*𝜃*_ = 4000) are used, whereas in (right) the asymptotic values *α*_*H*_ = 0.259 and *β*_*H*_ = 0.223 are considered. Three different panels are used in order to clearly show all the curves (color online)

An alternative to the use of *C*_*f*_ curves was explored by Sanmiguel Vila et al. [22], who employed the diagnostic-plot scaling [23] to assess the convergence to well-behaved conditions in ZPG TBLs. This scaling collapses data when the turbulence intensity, *u* ^′^/*U*, is plotted against the local mean velocity normalized with the edge velocity, *U*_*e*_. The apparent advantage of this scaling is that it is independent of indirectly measured quantities such as the wall-shear stress and the absolute wall distance, or other integral parameters, and is solely based on directly measured quantities, thereby excluding additional measurement uncertainties. It turns out that this plot exhibits an extended linear behavior in the outer region of ZPG TBLs (including the logarithmic region). In particular, this region follows the relation:

10 $$ \frac{u^{\prime}}{U}=\alpha_{d}-\beta_{d} \frac{U}{U_{e}}, $$

where *α*_*d*_ and *β*_*d*_ are fitting parameters. Note that the linear region extends with increasing *R* *e* towards lower *U*/*U*_*e*_-values [23]. Regarding PG TBLs, Drózdz et al. [48] and Vinuesa et al. [39] showed that the diagnostic scaling also collapsed boundary layers subjected to a wide range of pressure-gradient conditions when introducing the shape factor on the left-hand-side of relation (10) as: $u^{\prime } / \left (U \sqrt {H} \right )$. In Fig. 5 (left) and (middle) we show this scaling applied to several of the in-house APG TBLs considered in the present study, where it can be observed that despite the different pressure-gradient magnitudes and flow histories, all the profiles collapse in the outer region. In particular, a linear behavior is observed in the range 0.8 ≤ *U*/*U*_*e*_ ≤ 0.9 in all the cases. Note that the reduced range of the linear region is probably attributed to the relatively low *R* *e*-range considered here, since the higher- *R* *e* cases shown in Fig. 5 (right) exhibit longer regions of linear behavior. This observation will be exploited in the present work to define a criterion of convergence to well-behaved conditions, by inspecting the region of the boundary layer between *U*/*U*_*e*_ = 0.8 and 0.9, and fitting it to the relation:

11 $$ \frac{u^{\prime}}{U \sqrt{H}}=\alpha_{H}-\beta_{H} \frac{U}{U_{e}}, $$

where *α*_*H*_ and *β*_*H*_ are different fitting parameters valid for PG TBLs, when using the diagnostic plot scaled with *H*. The collapse observed in Fig. 5 motivates the development of another convergence criterion, valid for any APG TBL regardless of the flow history. The idea is to measure profiles of the streamwise mean velocity and streamwise velocity fluctuations, use the diagnostic-plot scaling modified with the shape factor, and assess whether they follow the linear behavior given by Eq. 11. Note that in general it is easier to perform profile measurements than to accurately measure wall-shear stress at a number of streamwise locations, and therefore this method has a wider range of applicability from an experimental point of view. Furthermore, the region adhering to Eq. 11 will extend with increasing *R* *e*, as observed in studies from ZPG TBLs [23].

**Fig. 6:**
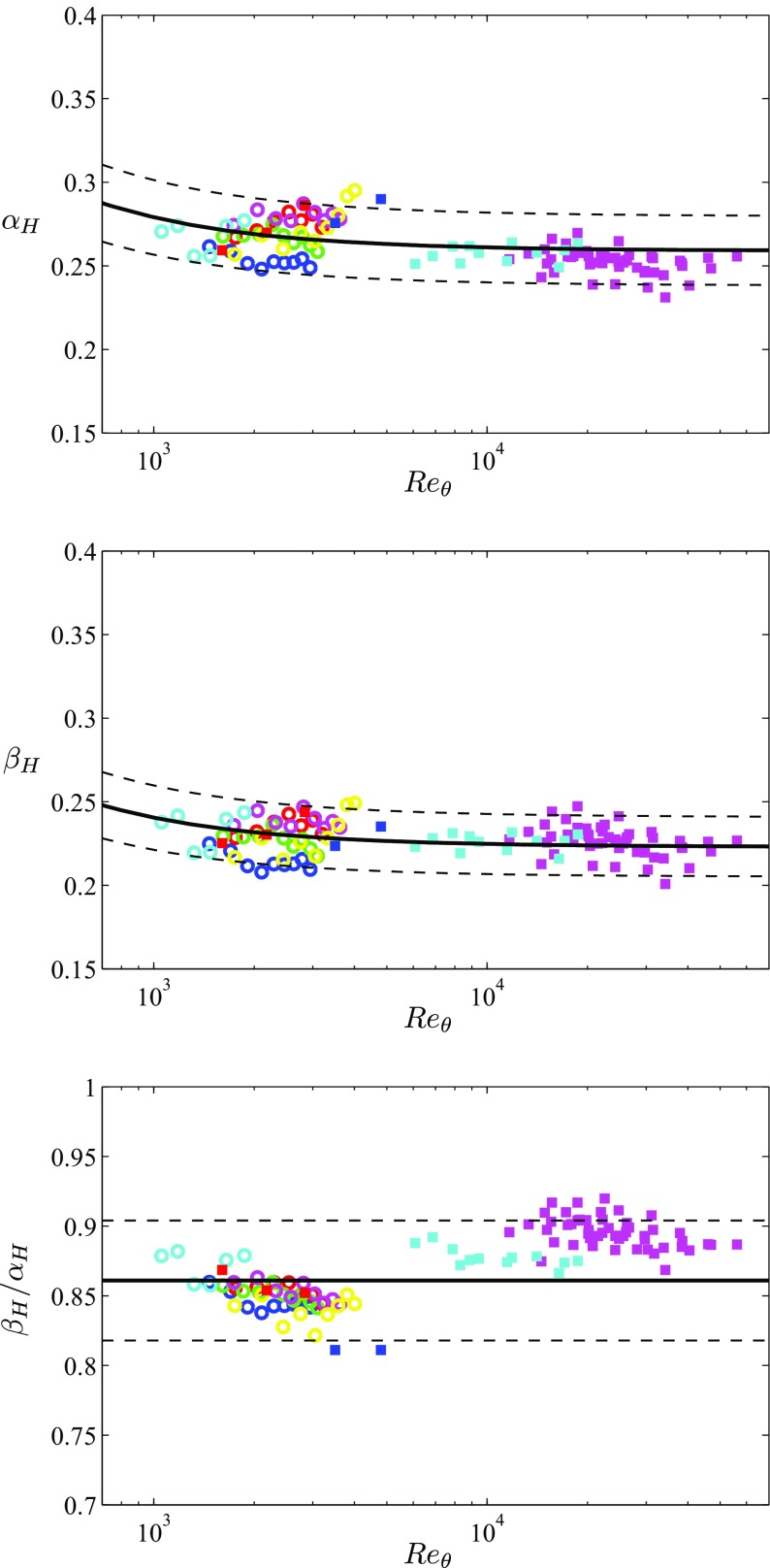
Evolution with *R* *e*_*𝜃*_ of the diagnostic-scaling parameters in Eq. 11, namely (top) *α*_*H*_, (middle) *β*_*H*_ and (bottom) their ratio *β*_*H*_/*α*_*H*_.  represents (top) Eq. 12 ± 8*%*, (middle) Eq. 13 ± 8*%* and (bottom) 0.861 ± 5*%*. Color code is given in Tables 1 and 2 (color online)

A convergence criterion can therefore be defined by using the in-house APG TBL data presented in this study, together with the numerical databases by Kitsios et al. [25] and Lee [26] and the experimental results by Monty et al. [27] and Nagib et al. [28]. The experimental results are used to define the present criterion in a more robust way, up to *R* *e*_*𝜃*_ = 18,700 and 56,100, respectively. The present criterion does not rely on the specific flow history, but on the local state of the boundary layer, which is manifested in the local value of the shape factor *H*. By fitting all the profiles to Eq. 11 in the linear region, approximately given by 0.8 ≤ *U*/*U*_*e*_ ≤ 0.9, one obtains the values of *α*_*H*_ and *β*_*H*_ shown in Fig. 6 for all the cases under consideration. The following empirical correlations can be used to describe the evolution with *R* *e*_*𝜃*_ of the coefficients *α*_*H*_ and *β*_*H*_:

12 $$ \alpha_{H}={ 0.259} + \frac{20}{Re_{\theta}}, $$

13 $$ \beta_{H}={ 0.223} + \frac{17.5}{Re_{\theta}}. $$

As also observed in Fig. 6 (bottom), the ratio *β*_*H*_/*α*_*H*_ shows an approximately constant value of around 0.861. Thus, a criterion based on calculating the values of *α*_*H*_ and *β*_*H*_, comparing them with the correlations (12) and (13), and with the ratio 0.861, can be defined. It is interesting to note that when considering the ZPG TBL data analyzed by Sanmiguel Vila et al. [22], and recomputing their ratio *β*_*H*_/*α*_*H*_, a value very close to the one found here, i.e. 0.861, is obtained. Note that the boundary layer by Spalart and Watmuff [16] also exhibits a linear region when represented in the diagnostic scaling following Eq. 11. However, due to the low Reynolds number and complicated flow history, this region was observed over a narrow range, and therefore the values of *α*_*H*_ and *β*_*H*_ from this profile were not included in Fig. 6. This is a similar method to the one proposed by Sanmiguel Vila et al. [22] for ZPG TBLs, with the novelty that it can be applied to general PG TBLs, regardless of their particular flow history. The advantage of using this criterion over the ones based on skin-friction curves is the fact that it only relies on velocity profile measurements, but not on direct measurements of *τ*_*w*_. Note, however, that these profile measurements require an accurate determination of the wall position, due to the fact that the shape factor *H* is necessary to obtain the diagnostic-plot scaling in relation (11). In the following section we will discuss the possibility of using another convergence criterion based on the diagnostic-plot scaling, which does not require an accurate determination of the wall position.

### Possibility of defining a criterion for cases with constant *β* and *x*-scans

Although the criterion presented in Section 4.1 represents an advantage with respect to the approaches based on *C*_*f*_ curves, it still requires the measurement of velocity profiles at a number of streamwise locations. In fact, since the shape factor has to be determined from these profiles, it is also necessary to obtain an accurate estimation of the wall position, which could be problematic especially in hot-wire anemometry measurements (see, for instance, the work by Örlü and Vinuesa [49]). This is the technique preferred when near-wall measurements are required. An alternative to the full profile measurements was proposed by Sanmiguel Vila et al. [22] for ZPG TBLs, consisting in *x*-scans in the outer region of the boundary layer. In this method, the probe is moved in the streamwise direction, measuring *u* ^′^ and *U*, and these values are then compared to Eq. 10 in order to assess the location of convergence towards canonical ZPG conditions. Unfortunately, the *x*-scan approach cannot be used with the method discussed in Section 4.1, due to the fact that it is necessary to measure the full profile in order to obtain *H*. On the other hand, the diagnostic scaling (10) applied to APG TBLs also leads to a linear region, in this case between 0.7 ≤ *U*/*U*_*e*_ ≤ 0.9 [39], although the particular values of *α*_*d*_ and *β*_*d*_ depend on the magnitude of the APG and the flow history. Therefore, as it was the case in Section 3.1, it is not possible to define a general criterion for any APG TBL, due to the fact that any flow history would produce a different evolution of *α*_*d*_ and *β*_*d*_ with *R* *e*_*𝜃*_.

As we did in Section 3.1, it is in principle possible to define reference curves for particular boundary layers with a prescribed flow history (for instance, for APG TBLs with constant values of *β*). Note that in this case the Reynolds-number evolution of *α*_*d*_ and *β*_*d*_ for a certain constant- *β* APG could also be used as a criterion to identify well-behaved TBLs. Thus, a probe can be located in the outer region of the boundary layer (in particular, in 0.7 ≤ *U*/*U*_*e*_ ≤ 0.9, which covers a large part of the boundary layer in terms of the boundary-layer thickness) and traversed horizontally in the streamwise direction to obtain measurements of *u* ^′^ and *U*. As in the work by Sanmiguel Vila et al. [22], these *x*-scans can then be compared with the values obtained from the empirical relations for *α*_*d*_ and *β*_*d*_, potentially producing a convergence criterion for wind-tunnel experiments of constant- *β* APG TBLs. However, additional databases spanning wider Reynolds-number ranges would be required to accurately define such a criterion.

## Conclusions

In the present work we analyze a total of six in-house APG TBLs and five additional high-quality databases of PG TBLs available in the literature [16, 25–28]. These TBLs, which exhibit very different albeit relatively simple flow histories, are used to define several criteria to assess the convergence of APG TBLs to well-behaved conditions. These criteria can be used for numerical databases, but are also useful in experimental design in order to assess if local effects in the wind tunnel have an impact on the development of the boundary layer. The assessment of whether a particular boundary layer can be considered as *well-behaved*, i.e., it is independent of the inflow conditions and is exempt of numerical or experimental artifacts, has received some attention in the past years. In particular, Chauhan et al. [5] proposed empirical fits for the shape factor *H* and the wake parameter π, and Sanmiguel Vila et al. [22] developed a method based on the diagnostic-plot scaling, all of them in the context of ZPG TBLs. In this article we aim at extending some of these criteria to APG TBLs, with the additional complexity introduced by the pressure gradient and thus the flow history. Note that it is simpler to define such criteria for the ZPG TBL, due to the fact that this flow case is uniquely defined by the Reynolds number. However, arbitrary *β*(*x*) evolutions can define specific PG TBLs, a fact that significantly complicates the possibility of establishing criteria for well-behaved PG TBLs. It must be highlighted that the criteria presented in this study have been developed using APG flows with simple *β*(*x*) evolutions, including constant, increasing and decreasing *β*(*x*) trends. Therefore, we proposed the following three convergence criteria, to be used depending on the particular boundary layer under consideration, as long as the flow exhibits a relatively simple flow history as discussed above:

1. *Empirical curves of* *C*_*f*_ and *H* for constant- *β* *APG TBLs.* These criteria are based on the empirical correlations (2) and (4) for *C*_*f*_ and *H*, respectively. The coefficients corresponding to the various cases under consideration are given in Table 3. The advantage of these criteria is that they are relatively easy to evaluate, although they require direct measurements of wall-shear stress and profile measurements with accurate wall position, respectively. Moreover, they cannot be used for APGs with general *β*(*R* *e*_*𝜃*_) distributions. The correlations given in the present work correspond to APG TBLs with constant values of *β* = 1 and 2. It is therefore apparent that a large database of constant- *β* PG TBLs needs to be compiled before general criteria for a wide range of *R* *e* and *β* conditions can be established.
2. *Empirical curve of* *C*_*f*_ for general *β*(*R* *e*_*𝜃*_) *PG TBLs.* This criterion is based on the observation that it is possible to obtain the *C*_*f*_ curve of any PG TBL by using only ZPG TBL data, and the *β*(*R* *e*_*𝜃*_) curve. The reference *C*_*f*_ curve of the particular PG boundary layer is obtained from Eq. 8, where *C*_*f*,ZPG_ and *H*_ZPG_ can be determined from the curves (2) and (4), and $\overline {\beta }$ is computed from relation (9). This is a very flexible method, with the only drawback of requiring direct measurements of the wall-shear stress at a number of locations, which may be impractical in wind-tunnel experiments.
3. *Diagnostic-plot scaling for general* *β*(*R* *e*_*𝜃*_) *PG TBLs.* This method relies on the collapse observed in the region 0.8 ≤ *U*/*U*_*e*_ ≤ 0.9 when using the diagnostic-plot scaling (modified with *H*), over a wide range of pressure-gradient conditions. The idea behind this criterion is to perform a linear fit to Eq. 11 in the region 0.8 ≤ *U*/*U*_*e*_ ≤ 0.9, and to compare the obtained values of *α*_*H*_ and *β*_*H*_ to the empirical curves (12) and (13). The only disadvantage of this method is the fact that it requires profile measurements at a number of locations, with an accurate determination of the wall position and the boundary-layer thickness (to compute *H*).

A fourth criterion, based on the diagnostic-plot scaling and *x*-scans, could be also defined for constant- *β* APG TBLs. This would be an extension of the idea proposed by Sanmiguel Vila et al. [22] of using *x*-scans to assess the convergence of ZPG TBLs. Given the empirical observation that APG TBLs exhibit a linear region following Eq. 10 for 0.7 ≤ *U*/*U*_*e*_ ≤ 0.9, it is in principle possible to measure values of *u* ^′^ and *U* at a particular height, and traverse the probe downstream within the outer layer in order to assess beyond which point they follow the Reynolds-number trend of *α*_*d*_ and *β*_*d*_ described by the proposed criterion. The approach with the *x*-scans would allow a more accurate determination of the point of convergence to well-behaved conditions.

This work constitutes a first step towards developing convergence criteria to evaluate whether a particular APG exhibits well-behaved conditions or not, when subjected to relatively simple *β*(*x*) evolutions. A first step in assessing the effect of PGs on TBLs consists of separating the effect of flow history and APG magnitude, as discussed by Bobke et al. [24], Monty et al. [27] and Vinuesa et al. [45]. Given the different flow histories exhibited by the boundary layers discussed in the present work (see Figs. 1 and 2), it is crucial to use robust criteria to ensure that the boundary layer under consideration is in fact subjected to the desired flow history. This is especially relevant in experimental studies, where it is usually not possible to obtain accurate measurements everywhere in the domain of interest, and therefore the methods described above may be quite relevant to ensure adequate flow conditions. Also note that although the specific applicability of some of the aforementioned criteria is limited to *β* = 0, 1 or 2, it should be noted that there is a lack of APG TBL data in the literature with significant regions of constant *β* and a documented flow history. Future work will be devoted to extend these criteria to obtain general expressions for *α*_*d*_ and *β*_*d*_ as a function of the APG magnitude. This will also require datasets up to higher Reynolds numbers, over a wider range of constant- *β* values. In addition to these, additional high-quality numerical and experimental studies of PG TBLs with more complex flow histories, at sufficiently high Reynolds numbers, are required to test and extend the criteria presented in this study. Note that, although these criteria provide robust results in the APG cases with relatively simple flow histories under study here, they may not be accurate when considering more complex *β*(*x*) evolutions. Therefore the methods described here should be considered as a first attempt to establish criteria to identify well-behaved APGs.
